# Инициация пубертата гонадотропинами у мальчиков с врожденным изолированным гипогонадотропным гипогонадизмом

**DOI:** 10.14341/probl13141

**Published:** 2023-02-25

**Authors:** К. Д. Кокорева, И. С. Чугунов, М. А. Карева, О. Б. Безлепкина

**Affiliations:** Национальный медицинский исследовательский центр эндокринологии; Национальный медицинский исследовательский центр эндокринологии; Национальный медицинский исследовательский центр эндокринологии; Национальный медицинский исследовательский центр эндокринологии

**Keywords:** врожденный гипогонадотропный гипогонадизм, синдром Кальмана, комбинированная заместительная гормональная терапия, хорионический гонадотропин, рекомбинантный фолликулостимулирующий гормон

## Abstract

**ОБОСНОВАНИЕ:**

ОБОСНОВАНИЕ. Терапия гонадотропинами у мальчиков с врожденным изолированным гипогонадотропным гипогонадизмом в сравнении с препаратами тестостерона позволяет добиться увеличения объема яичек и индуцировать сперматогенез. Однако сложность титрации дозы, неполный ответ на терапию, особенно при врожденных формах заболевания, отсутствие общепринятых протоколов ведения не позволяют широко использовать данный метод терапии в отношении индукции пубертата у подростков с синдромом Кальмана или нормоосмическим вариантом гипогонадотропного гипогонадизма.

**ЦЕЛЬ:**

ЦЕЛЬ. Оценка эффективности комбинированной гормональной заместительной терапии препаратами хорионического гонадотропина (ХГ) и рекомбинантного фолликулостимулирующего гормона (рФСГ) в отношении индукции пубертата у подростков с врожденным изолированным нормоосмическим гипогонадотропным гипогонадизмом и синдромом Кальмана.

**МАТЕРИАЛЫ И МЕТОДЫ:**

МАТЕРИАЛЫ И МЕТОДЫ. Одноцентровое проспективное открытое неконтролируемое исследование. В течение 12 мес мальчики с гипогонадотропным гипогонадизмом получали заместительную терапию гонадотропинами: исходные дозы препаратов ХГ составили 500 МЕ в неделю, рФСГ — 37,5 МЕ в неделю, через 6 мес терапии дозы препаратов были двукратно увеличены. У всех пациентов оценивались антропометрические данные, динамика полового развития, объем тестикул, уровни тестостерона, ингибина В, антимюллерова гормона (АМГ) до инициации пубертата и через 2, 6 и 12 мес терапии.

**РЕЗУЛЬТАТЫ:**

РЕЗУЛЬТАТЫ. В исследование были включены 8 подростков с гипогонадотропным гипогонадизмом, медиана возраста перед началом терапии составила 15,7 года [15,33; 16,41]. Через 12 мес лечения гонадотропинами у 7 из 8 пациентов отмечались изменения со стороны мошонки и полового члена до стадии G2–3. На фоне лечения отмечалось увеличение медианы уровня тестостерона с 0,44 [0,34; 0,62] до 4,39 нмоль/л [0,88; 10,51] (p=0,012), снижение уровня АМГ с 35,70 [18,00; 59,00] до 14,41 нг/мл [11,60; 16,65] (p=0,017). Объем тестикул и уровень ингибина В статистически значимо не изменились.

**ЗАКЛЮЧЕНИЕ:**

ЗАКЛЮЧЕНИЕ. Терапия гонадотропинами является эффективным методом инициации пубертата у подростков с врожденным гипогонадотропным гипогонадизмом, позволяющим добиться не только андрогенизации, но и созревания клеток Сертоли.

## Abstract (en)

**BACKGROUND:**

BACKGROUND: Gonadotropin therapy in boys with congenital isolated hypogonadotropic hypogonadism helps to increase testes volume and induce spermatogenesis in comparison with testosterone therapy. However, difficulties with dose titration, partial therapy success, absence of generally accepted regimen protocols don’t allow to use this therapy in order to induce puberty in adolescents with Kallmann syndrome or normosmic hypogonadotropic hypogonadism.

**AIM:**

AIM: To assess the effectiveness of combination hormonal replacement therapy via human chorionic gonadotropin and recombinant follicle stimulation hormone in adolescents with congenital isolated normosmic hypogonadotropic hypogonadism and with Kallmann syndrome

**MATERIALS AND METHODS:**

MATERIALS AND METHODS: This is an open single-center prospective non-controlled study. Boys with hypogonadotropic hypogonadism were receiving hormonal replacement therapy for 12 months. Initial dose of human chorionic gonadotropin was 500 IU per week. Initial dose of recombinant follicle stimulation hormone was 37.5 IU per week. Doses were doubled in 6 months. Antropometric data, Tanner stage, testes volumes, inhibin B and anti-Mullerian hormone (AMH) levels were evaluated in all the patients before the treatment, after 6 and 12 months of the therapy.

**RESULTS:**

RESULTS: 8 boys with hypogonadotropic hypogonadism were included into the study. Median age before therapy initiation was 15.7 years [15.33; 16.41]. In 12 months after the therapy initiation puberty development, testosterone increase from 0.44 [0.34;0.62] to 4.39 [0.88;10.51] nmol/l (p=0.012), AMH decrease from 35.70 [18.00;59.00] to 14.41 [11.60;16.65] ng/ml were noted in all the patients (p=0.017). Testes volumes increase and inhibin B level increase were not statistically significant.

**CONCLUSION:**

CONCLUSION: Gonadotropin therapy is effective in order to puberty initiation in adolescents with congenital hypogonadotropic hypogonadism. In helps to achieve not only androgenization, but also to Sertoli cells maturation.

## ОБОСНОВАНИЕ

Гипогонадотропный гипогонадизм представляет собой гетерогенную группу заболеваний, ассоциированных с нарушением секреции и действия гонадотропин-рилизинг-гормона (ГнРГ) и гонадотропинов при сохранной функции гонад. Приобретенный гипогонадотропный гипогонадизм встречается чаще и развивается у детей при объемных образованиях хиазмально-селлярной области (краниофарингиомы, глиомы, аденомы гипофиза и др.). Врожденные формы заболевания встречаются редко (от 1:10 000 до 1:30 000 новорожденных мальчиков и до 1:120 000 среди девочек), а их развитие ассоциировано с вариантными заменами в более чем 40 генах [1]. В большинстве случаев врожденный гипогонадотропный гипогонадизм наследуется по аутосомно-доминантному типу, однако описаны аутосомно-рецессивный и Х-сцепленный типы наследования, олигогенный характер имеют до 20% случаев заболевания.

В настоящее время у взрослых пациентов с гипогонадотропными формами гипогонадизма лишь в половине случаев удается добиться фертильности, в основном это касается пациентов с приобретенными формами гипогонадизма. Традиционно для лечения гипогонадизма у мальчиков, как первичного, так и вторичного, используются препараты тестостерона. Данная терапия позволяет добиться необходимой андрогенизации пациентов, но объем гонад остается допубертатным, и созревания сперматогенного эпителия не происходит [2].

Альтернативным методом индукции пубертата у подростков является применение препаратов хорионического гонадотропина человека (ХГч) и рекомбинантного фолликулостимулирующего гормона (рФСГ). Раннее применение препаратов рФСГ позволяет добиться дифференциации клеток Сертоли и увеличения объема яичек в пубертате [3][4]. Созревание сперматогенного эпителия под действием препаратов гонадотропинов в подростковом возрасте повышает вероятность фертильности во взрослом возрасте [5].

Под влиянием лютеинизирующего гормона (ЛГ) или гомологичного ему по механизму действия ХГч внутритестикулярная концентрация тестостерона, синтезируемого клетками Лейдига, повышается до 40 раз, значимо превышая концентрацию тестостерона в крови. Паракринное влияние тестостерона совместно с ФСГ на клетки Сертоли стимулирует их созревание, о чем косвенно можно судить по увеличению объема гонад и изменению уровня АМГ, в то время как введение тестостерона извне не позволяет добиться повышения внутритестикулярной концентрации тестостерона [6][7].

Несмотря на вышеперечисленные преимущества терапии гонадотропинами, такая терапия имеет свои ограничения: большая кратность введения препаратов, сложность титрации дозы, неполный ответ на терапию, особенно при врожденных формах заболевания [8]. До настоящего времени не определены дозы и режим введения ХГч и рФСГ, являющиеся оптимальными для инициации заместительной терапии у мальчиков-подростков с гипогонадотропным гипогонадизмом [9][10].

## ЦЕЛЬ ИССЛЕДОВАНИЯ

Оценка эффективности комбинированной гормональной заместительной терапии препаратами ХГч и рФСГ в отношении индукции пубертата у подростков с врожденным изолированным нормоосмическим гипогонадотропным гипогонадизмом и синдромом Кальмана.

## МАТЕРИАЛЫ И МЕТОДЫ

Место и время проведения исследования

Место проведения. Исследование проведено на базе Института детской эндокринологии ФГБУ «НМИЦ эндокринологии» Минздрава России.

Продолжительность исследования. Исследование проводилась в рамках клинического исследования «Апробация метода инициации пубертата с помощью гонадотропных препаратов у мальчиков с гипогонадотропным гипогонадизмом». Длительность исследования составила 12 мес.

Изучаемые популяции (одна или несколько)

Популяция: 8 мальчиков с изолированным врожденным гипогонадотропным гипогонадизмом. Диагноз гипогонадотропного гипогонадизма был установлен на основании задержки полового развития или ареста пубертата на срок более 1 года, низких базальных показателей ЛГ, тестостерона, низких стимулированных уровней ЛГ (менее 10 мМЕ/мл) на фоне пробы с аналогом гонадолиберина.

Критерии включения: возраст от 14 лет и старше, мужской пол, диагноз «изолированный врожденный гипогонадотропный гипогонадизм/синдром Кальмана», показатели костного возраста более 12 лет, подписание информированного согласия на участие в исследовании.

Критерии исключения: множественный дефицит гормонов гипофиза, отказ от участия в исследовании.

Критерии прекращения участия в исследовании: возникновение клинически значимых, острых или обострение хронических заболеваний сердечно-сосудистой, нервной, мочеполовой систем, желудочного-кишечного тракта и заболеваний крови.

Способ формирования выборки из изучаемой популяции (или нескольких выборок из нескольких изучаемых популяций)

Сплошной способ формирования выборки.

Дизайн исследования

Одноцентровое интервенционное динамическое проспективное одновыборочное неконтролируемое исследование. Пациенты наблюдались в течение 1 года (визиты перед началом исследования, через 2, 6 и 12 мес исследования).

Описание медицинского вмешательства (для интервенционных исследований)

Все пациенты получали сочетанную терапию препаратами ХГч (лиофилизат для приготовления раствора для внутримышечного введения, ФГУП «Московский эндокринный завод») и рФСГ (ГОНАЛ-ф, ООО «Мерк», подкожные инъекции) в течение одного года: доза ХГч в течение первых 6 мес терапии составляла 500 МЕ 1 раз в неделю с дальнейшим увеличением до 1000 МЕ в неделю, доза рФСГ составляла 37,5 МЕ 1 раз в неделю с дальнейшим увеличением до 75 МЕ 1 раз в неделю.

Методы

Протокол наблюдения включал проведение физикального обследования с антропометрией (оценивались рост, масса тела, сегменты тела). Инструментальная диагностика включала УЗИ органов мошонки перед началом терапии, через 2, 6 и 12 мес лечения, рентгенографию кистей рук с оценкой костного возраста перед началом лечения и по его окончании. Лабораторная диагностика включала исследование уровней тестостерона, ингибина В и АМГ перед началом лечения, через 2, 6 и 12 мес терапии.

При включении в исследование и на визитах через 6 и 12 мес от инициации терапии проводилась оценка физического развития, которая включала определение показателей роста, массы тела, индекса массы тела. Показатели роста и массы тела оценивались по перцентильным таблицам Cole Т. и соавт. [11] для данного пола и возраста. Расчет показателей SDS роста, массы тела, индекса массы тела, сегментов тела производился с помощью компьютерной программы Auxology 1,0 b17 (Pfizer, США).

Для оценки полового развития использовалась классификация Таннера, объем яичек оценивался с помощью орхидометра Прадера.

Концентрации ЛГ, ФСГ перед началом терапии и тестостерона базально и на фоне лечения через 2, 6 и 12 мес оценивались методом усиленной хемилюминесценции с помощью автоматического иммунохимического анализатора Vitros 3600 (Ortho Clinical Diagnostics, Johnson& Johnson, США).

Перед началом терапии всем пациентам была проведена проба с аналогом ГнРГ (бусерелин 300 мкг интраназально) с оценкой уровней ЛГ и ФСГ на 0, 60 и 240-й минутах теста.

Измерение уровня ингибина В (Inhibin B Gen II ELISA) и АМГ (AMH Gen II ELISA) проводилось методом иммуноферментного анализа с помощью наборов компании Beckman Coulter (Beckman Coulter, Inc., США).

Оценка концентрации тестостерона на фоне лечения проводилась через 48 ч от инъекции препаратов ХГч и рФСГ. Все гормональные исследования осуществлялись в клинико-диагностической лаборатории ФГБУ «НМИЦ эндокринологии» Минздрава России.

Ультразвуковое исследование органов мошонки проводилось на ультразвуковом сканере (Voluson E8, GE Healthcare, Австрия) с использованием линейного датчика с частотой 10–12 Мгц. Объем тестикул рассчитывался, используя формулу объема эллипсоида:

0,52 × d1 × d2 × d3,

где d1, d2, d3 — переднезадний, верхненижний размеры яичка и толщина. Исследование интратестикулярного кровотока проводилось методом цветной допплерографии.

Костный возраст оценивался путем проведения рентгенографии кистей рук и лучезапястных суставов в прямой проекции до начала терапии и через 12 мес терапии по стандартной методике, определение костного возраста проводилось по рентгенологическому атласу W. Greulich и S. Pyle.

Магнитно-резонансная томография головы проводилась на аппарате Magnetom Harmony (Siemens, Германия) c напряженностью магнитного поля 1,0 Тесла. Исследование проводилось в Т1- и Т2-взвешенных режимах по стандартной методике с оценкой состояния обонятельных луковиц.

Наличие аносмии оценивалось субъективно путем прямого опроса пациента.

Молекулярно-генетический анализ проводился в лаборатории отделения наследственных эндокринопатий ФГБУ «НМИЦ эндокринологии» Минздрава России методом таргетного секвенирования следующего поколения (NGS) с применением авторской панели «Гипогонадотропный гипогонадизм» (технология Ion Ampliseq™ Custom DNA Panel, Thermo Scientific, Waltham, MA, USA), содержащей праймеры для мультиплексной ПЦР и секвенирования кодирующих последовательностей следующих 30 генов: FGF17, FGF8, CHD7, TAC3, PROK2, DNMT3L, DUSP6, FGFR1, FLRT3, GNRH1, GNRHR, HS6ST1, IL17RD, INSL3, ANOS1, KISS1, KISS1R, LHB, NSMF, POLR3B, PROKR2, RBM28, SEMA3A, SPRY4, TACR3, WDR11, GREAT, NR0B1, POLR3A, MKRN3. После анализа полученных данных проводилось подтверждение полученных мутаций методом Сэнгера на секвенаторе Genetic Analyzer Model 3130 (Thermo Scientific, Waltham, MA, USA). Для определения цис- или транс-положения пар гетерозиготных мутаций использовались данные обследования родителей, графический анализ прочтений BAM-файлов, а также TA-клонирование продуктов ПЦР с последующим секвенированием клонов методом Сэнгера. Оценка патогенности вариантов нуклеотидной последовательности проводилась согласно международным и российским рекомендациям.

Статистический анализ

Для выявления статистически значимых различий между двумя зависимыми группами был использован критерий Вилкоксона. Уровень р<0,05 считался статистически значимым. Расчет производился с помощью статистического пакета Statistica 12 (StatSoft inc., США).

Этическая экспертиза

Проведение исследования одобрено локальным этическим комитетом ФГБУ «НМИЦ эндокринологии» Минздрава России, Протокол № 18 от 11/10/2020).

Нежелательные явления

В ходе исследования нежелательных явлений зафиксировано не было.

## РЕЗУЛЬТАТЫ

В исследование включены 8 подростков с гипогонадотропным гипогонадизмом, медиана возраста пациентов на начало терапии составила 15,68 года [ 15,33; 16,41].

По данным клинического осмотра перед началом терапии мальчики имели нормальные росто-весовые показатели: медиана SDS роста -0,56 [-1,74; -0,08], медиана SDS индекса массы тела составила 0,84 [-0,86; 1,48]. Диспропорция сегментов тела наблюдалась у половины пациентов — отмечалось увеличение нижнего сегмента тела относительно верхнего. Все пациенты имели стадию G1 полового созревания, стадия пубархе P1 отмечалась у 3 из 8, у остальных 5 пациентов отмечалась стадия лобкового оволосения P2. У 3 пациентов (у пациентов 1, 2 и 6) отмечался при рождении крипторхизм (у 1, 6 — двусторонний, у 2 — односторонний), у 3 (пациентов 3, 7 и 8) отмечалась микропения.

Все пациенты имели допубертатные базальные уровни гонадотропинов (уровень ЛГ у всех пациентов составил менее 0,22 Ед/л, ФСГ менее 0,66 Ед/л) и тестостерона (медиана 0,43 [ 0,34; 0,62] нмоль/л). По результатам пробы с аналогом ГнРГ медиана максимального подъема ЛГ составила 1,68 Ед/л [ 1,00; 2,13], а ФСГ — 3,39 Ед/л. Медианы показателей ингибина B и АМГ перед началом лечения составляли 46,3 [ 16,95; 79,95] пг/мл и 35,7 [ 18,00; 59,00] нг/мл соответственно, то есть показатели находились в низконормальном диапазоне. У троих пациентов отмечались высокие значения ингибина B (более 65 пг/мл), что на этапе включения в исследование могло бы быть интерпретировано, скорее, как проявление конституциональной задержки пубертата, однако низкий уровень ЛГ на пробе с аналогом ГнРГ свидетельствовал о наличии у этих пациентов гипогонадизма.

По данным рентгенографии кистей рук костный возраст у всех отставал от паспортного, медиана костного возраста составила 13,5 [ 13,25; 13,50] года.

Объем тестикул по данным УЗИ до включения в исследование составлял 0,85 [ 0,59; 0,95] мл и соответствовал допубертатным значениям у всех пациентов.

Всем пациентам было проведено молекулярно-генетическое исследование. Вариантные замены в генах, входящих в молекулярно-генетическую панель, выявлены у 5 из 8 мальчиков: у 1 пациента выявлена мутация в гене FGFR1, у 1 — FGF8, у 1 — WDR11. У 2 пациентов были выявлены мутации в нескольких генах одновременно: в генах GNRHR и FGFR1 и в генах FGFR1 и SPRY4 (табл. 1). Таким образом, олигогенное наследование подтверждено в 2 случаях из 8.

Жалобы на сниженное восприятие запахов предъявляли 5 пациентов, по данным МРТ головного мозга гипоплазия обонятельных луковиц наблюдалась у 4 из них (табл. 1). У одного пациента жалобы на аносмию не сопровождались гипоплазией обонятельных луковиц по данным МРТ.

Результаты лечения

Все пациенты получали лечение в течение 1 года: первые 6 мес препаратами ХГч (500 МЕ в неделю) в сочетании с рФСГ (37,5 МЕ в неделю), спустя 6 мес дозы обоих препаратов были увеличены в 2 раза.

Отклонений от проведения процедур протокола, серьезных нежелательных явлений, эпизодов тяжелых побочных и аллергических реакций на введение гонадотропных препаратов, внеплановых госпитализаций по экстренным показаниям, обострений сопутствующей патологии или острых состояний, потребовавших досрочного выбывания, в группе наблюдения не было зарегистрировано.

Медиана скорости роста составила 4,79 см в год [ 3,95; 6,55], медиана SDS скорости роста — 2,83 SD [ 2,08; 3,46]. При этом диспропорция сегментов тела до лечения отмечалась у 4 пациентов, а через 12 мес лечения — у 6 из 8 пациентов. У пациента 2 отмечалась наиболее выраженная диспропорция сегментов тела (SDS верхнего сегмента -2,67 SD, SDS нижнего сегмента 1,35 SD), при этом по данным молекулярно-генетического исследования изменений выявлено не было, но с рождения отмечался односторонний крипторхизм. Наименее выраженная диспропорция сегментов тела определялась у пациента 4 (SDS верхнего сегмента -1,64 SD, SDS нижнего сегмента 0,47 SD).

Значимой прогрессии костного возраста на фоне проводимого лечения не отмечалось: медиана костного возраста до лечения составила 13,5 [ 13,25; 13,50], через 12 мес терапии — 13,5 [ 13,5; 14,25].

У подавляющего большинства подростков (у 7 из 8) наблюдалось прогрессирование полового развития (G) (рис. 1): у 4 пациентов — со стадии G1 до стадии G2 (увеличение полового члена, отвисание мошонки, изменение структуры мошонки и ее пигментирование), у 3 — до стадии G3 (дальнейшее увеличение и утолщение полового члена), у 1 пациента изменений со стороны мошонки и полового члена не отмечалось, при этом у пациента перед началом терапии наблюдалась прогрессия пубархе до стадии P2. Через год терапии прогрессия пубархе (P) наблюдалась у 3 пациентов: у двух пациентов со стадии P1 до стадии P2 и у одного до стадии P3 (рис. 1).

Достоверного увеличения размеров тестикул по результатам УЗИ на фоне проводимого лечения (рис. 2) не отмечалось: медиана объема яичек до лечения составила 0,85 [0,59; 0,95] и 0,81 [0,61; 1,85] мл после лечения, p=0,26.

На фоне лечения отмечалось достоверное увеличение уровня тестостерона: показатели тестостерона на визите 0 составляли 0,43 [0,34; 0,62], на визите 2 — 0,67 [0,475; 1,19], на визите через 6 мес — 1,01 [0,60; 5,36], через 12 — 4,39 [0,88; 10,51] нмоль/л (рис. 3, p=0,012). Через 6 мес лечения наиболее высокий уровень тестостерона определялся у пациента 8 и составлял 9,6 нмоль/л, у пациента 4 уровень тестостерона составил 8,8 нмоль/л, а у остальных пациентов показатели тестостерона оказались не выше 2 нмоль/л. Через 12 мес сочетанной терапии гонадотропинами наиболее высокий уровень тестостерона также определялся у пациента 4 (25,5 нмоль/л) и у пациента 8 (13,5 нмоль/л). При этом у пациента 8, у единственного из всей группы, не наблюдалось изменений со стороны мошонки и полового члена на фоне терапии. Самые низкие уровни тестостерона через 12 мес терапии определялись у пациентов 6 и 7, составив 0,3 и 0,5 нмоль/л соответственно. У остальных пациентов показатели тестостерона находились в диапазоне 1,2–7,6 нмоль/л, что соответствовало их стадии полового развития.

Уровень ингибина В на фоне лечения достоверно не увеличился: до лечения он составлял 46,30 [ 16,95; 79,95], через 12 мес терапии — 53,30 [ 40,85; 109,50] пг/мл (рис. 4, p=0,12). Наиболее низкие показатели ингибина B до терапии определялись у пациентов 1 и 2, которые являются родными братьями (13,6 и 10,0 пг/мл соответственно). Наиболее высокие показатели ингибина B до терапии определялись у пациентов 4 и 8 (81,5 пг/мл). Наиболее высокий уровень ингибина B после 12 мес терапии определялся у пациента 3 (153,3 пг/мл), наименьший уровень ингибина B через год терапии — у пациента 2 (23,4 пг/мл).

В нашем исследовании у пациентов отмечался низкий базальный уровень АМГ (Me 35,7 нг/мл). В первые 6 мес терапии уровень АМГ оставался на сопоставимом уровне с показателями АМГ до лечения. Значительное снижение уровня АМГ наблюдалось через полгода от начала терапии без периодов подъема. Через 12 мес лечения гонадотропинами у пациентов отмечалось достоверное снижение АМГ с 35,7 [ 18,0; 59,0] до 14,4 [ 11,6; 16,7] нг/мл (p=0,017, рис. 5), что свидетельствует о пролиферации и дифференциации клеток Сертоли.

Нежелательные явления на фоне терапии не наблюдались ни у одного из пациентов.

**Table:** Таблица 1. Результаты молекулярно-генетического исследования, некоторые фенотипические и базальные клинико-лабораторные характеристики, МРТ-оценка гипоплазии обонятельных луковиц пациентов

1 Д.А.	Не выявлены	Да	Да	Крипторхизм	0,4	21,6	13,6	1,0
2 М.А.	Не выявлены	Да	Да	Крипторхизм	0,4	13,4	10,0	1,2
3 А.И.	GNRHR c.785G>A p.R262Q;FGFR1 c.742G>A p.V248M	Нет	Нет	Микропения	0,6	32,6	20,3	0,6
4 И.О.	FGF8 c.77C>T p.P26L	Нет	Нет	Норма	0,6	70,0	81,5	0,9
5 И.П.	FGFR1 c.296A>G:p.Y99C;SPRY4 c.296A>G:p.Y99C	Да	Нет	Норма	0,6	14,4	78,4	0,9
6 В.Д.	Не выявлены	Нет	Нет	Крипторхизм	0,3	38,8	40,7	0,5
7 В.С.	FGFR1 c.1097C>T:p.P366L	Да	Да	Микропения	0,4	48,1	51,9	0,4
8 А.А.	WDR11 c.2932A>T p.K978X	Да	Да	Микропения	0,3	70,0	81,5	0,9

**Figure:**
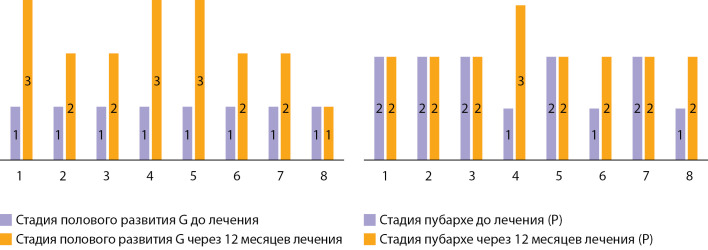
Рисунок 1. Половое развитие до и через 12 месяцев лечения.

**Figure:**
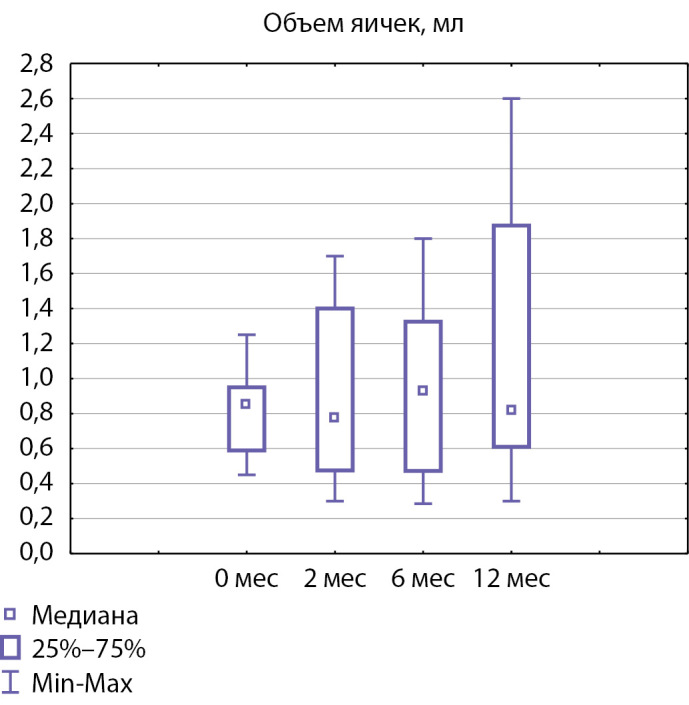
Рисунок 2. Медиана объема яичек до начала терапии и через 2, 6 и 12 месяцев лечения.

**Figure:**
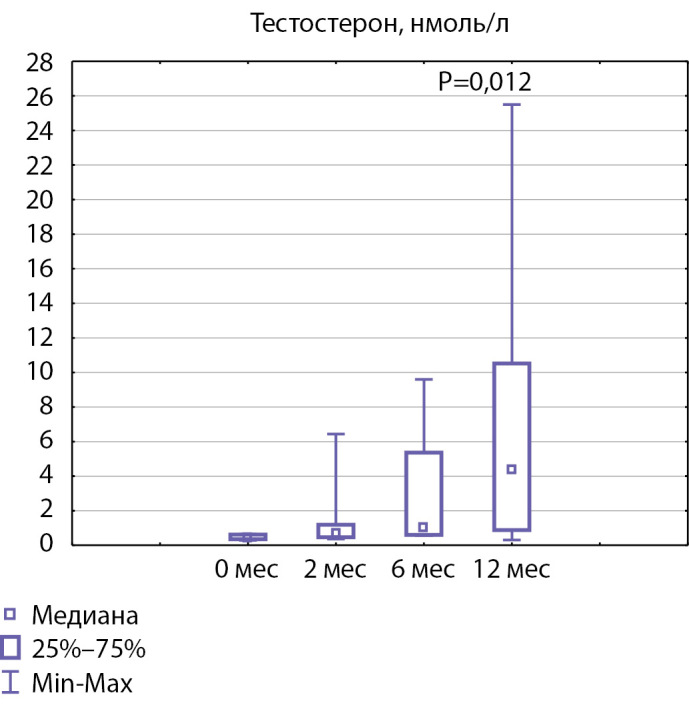
Рисунок 3. Медиана показателей тестостерона до начала терапии и через 2,6 и 12 месяцев лечения (p<0,05).

**Figure:**
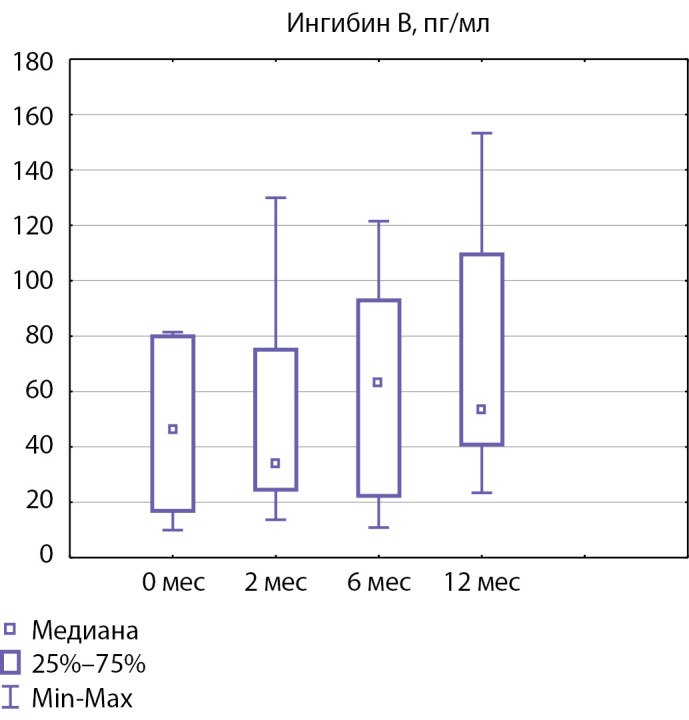
Рисунок 4. Медиана уровня ингибина B до начала терапии и через 2, 6 и 12 месяцев терапии.

**Figure:**
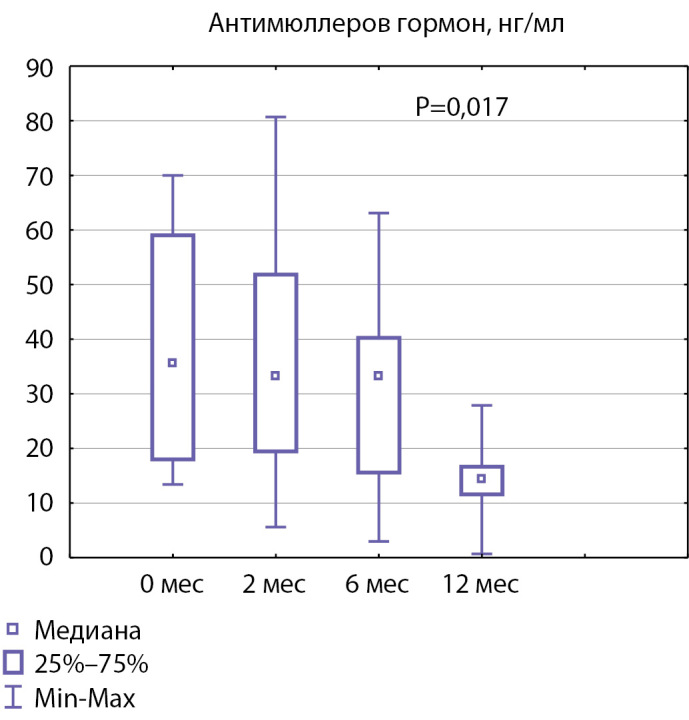
Рисунок 5. Медиана уровня АМГ до начала терапии и через 2, 6 и 12 месяцев терапии (p<0,05).

## ОБСУЖДЕНИЕ РЕЗУЛЬТАТОВ

Вовремя инициировать половое развитие и предупредить формирование диспропорции сегментов тела возможно при своевременном начале заместительной терапии. В отсутствие крипторхизма и микропении в периоде детства поставить данный диагноз затруднительно. В нашем исследовании крипторхизм определялся у 3 пациентов, микропения также у 3, сочетания крипторхизма и микропении не наблюдалось ни у кого из пациентов, чем, вероятнее всего, объясняется позднее обращение к эндокринологу и позднее установление диагноза.

Сочетание гипогонадизма с аносмией наблюдалось у 5 пациентов из 8, однако не у всех пациентов с аносмией определялась гипоплазия обонятельных луковиц по результатам МРТ головного мозга. Подобное наблюдение в 2021 г. было описано V. Danda и соавт., которые отметили, что степень нарушения обоняния не во всех случаях коррелирует с гипоплазией луковиц по данным МРТ [12].

Всем пациентам проводилось молекулярно-генетическое исследование 30 генов, вариантные замены в которых могут приводить к развитию гипогонадотропного гипогонадизма. По данным зарубежных авторов, вариантные замены выявляются в 30–50% случаев гипогонадизма [1]. В нашем исследовании клинически значимые вариантные замены были выявлены у 5 из 8 пациентов (62,5%). Наиболее часто выявлялись дефекты в гене FGFR1 (у 3 из 8 пациентов, т.е. у 37,5% пациентов). У 2 пациентов вариантные замены в гене FGFR1 были выявлены в составе дигенных нарушений вместе с дефектами в генах GNRHR и SPRY4. Несмотря на то что в разных работах поиск дефектов проводится в различных группах генов, а также несмотря на малое количество пациентов в нашем исследовании, полученные результаты согласуются с данными о том, что дефекты гена FGFR1, наряду с дефектами в гене GNRHR, при гипогонадизме у мальчиков выявляются наиболее часто, и особенно часто — в составе дигенных нарушений [13–15].

К основным клиническим проявлениям гипогонадизма относят задержку полового развития и, как следствие, формирование диспропорции сегментов тела. В настоящее время для инициации пубертата используются разные дозы ХГч (350–1000 МЕ) и различные режимы введения (1–2 раза в неделю), однако степень прогрессии полового развития и повышение уровня тестостерона очень вариабельны и в значительной степени зависят от внутриутробной стимуляции гонад. Перед началом нашего исследования диспропорция отмечалась у 4 мальчиков из 8, а к концу лечения — у 6. Возможно, одной из причин формирования диспропорции сегментов тела стало использование низких доз ХГч на первом этапе инициации пубертата, что привело к недостаточной выработке тестостерона и формированию евнухоидных пропорций тела вследствие медленного закрытия зон роста. Наличие диспропорции сегментов еще до начала лечения, по-видимому, может быть одним из критериев в пользу инициации у таких пациентов монотерапии тестостероном или в комбинации с ХГч. Однако, по данным ретроспективного анализа данных 19 подростков и молодых взрослых с врожденным гипогонадотропным гипогонадизмом, формирование диспропорции сегментов у таких пациентов не зависит от дозы получаемых препаратов ХГч, но зависит от хронологического и костного возраста, в котором терапия была инициирована [16].

Как видно из рисунка 1, прогрессия полового развития до стадии G3 отмечалась у пациентов №1, 4, 5, у пациентов №2, 3, 6 и 7 — до стадии 2, у пациента 8 прогрессии полового развития не наблюдалось. Результаты нашего исследования согласуются с результатами зарубежных исследований: по данным исследования С. Gong и соавт. с участием 22 подростков с изолированным гипогонадотропным гипогонадизмом, применение препаратов ХГч позволило у большинства из них добиться прогрессии полового развития до стадии Tanner 2, у некоторых — до стадии 3 [17]. Как и в нашем исследовании, в исследовании С. Gong и соавт. были пациенты, добиться прогрессии полового развития у которых не удалось, что, вероятно, объясняется недостаточным ответом на терапию у пациентов с полными формами гипогонадизма [17]. С другой стороны, длительное применение гонадотропинов даже у пациентов с тяжелыми врожденными формами заболевания может позволить не только увеличить объем гонад, но и индуцировать сперматогенез [18]. По данным исследования D. Swee и соавт., появление сперматогенеза само по себе повышает качество жизни пациентов с гипогонадизмом даже при неудовлетворительных результатах спермограммы, увеличивая ощущение «мужественности» [19]. В нашем исследовании спермограмма у подростков не исследовалась, а для оценки созревания клеток Сертоли использовались такие показатели, как ингибин B и АМГ. Ингибин B продуцируется клетками Сертоли под действием ФСГ, выступая в роли регулятора секреции этого гормона по механизму отрицательной обратной связи. Уровень ингибина B изменяется в течение жизни мужчины: в младенчестве определяется высокий уровень ингибина B, затем, к возрасту 6–10 лет, он снижается до низких значений, впоследствии увеличиваясь и достигая пика к 12–17 годам [20].

АМГ — димерный гликопротеин, принадлежит к семейству трансформирующих факторов роста. Внутриутробно АМГ продуцируется клетками Сертоли в яичках плода и отвечает за регрессию мюллеровых протоков. В течение первых месяцев жизни мальчика уровень АМГ возрастает, достигая наибольших значений в 6 мес. Затем медленно снижается [21]. Уровень АМГ считается маркером ФСГ-стимулированной пролиферации клеток Сертоли. Также обсуждается роль АМГ в качестве предиктора сперматогенной активности [22].

Известно, что у мальчиков с гипогонадотропным гипогонадизмом до начала терапии определяются низкие показатели как ингибина В, так и АМГ, что связано с недостаточной активностью клеток Сертоли в период мини-пубертата [23]. Терапия препаратами ХГч у таких пациентов приводит к более выраженному снижению уровня АМГ, чем у пациентов, получающих терапию тестостероном, несмотря на сопоставимые уровни тестостерона в крови. Это объясняется повышенной концентрацией внутритестикулярного тестостерона у пациентов на терапии препаратами ХГч: более высокая концентрация внутритестикулярного тестостерона полностью ингибирует секрецию АМГ в гонадах [24]. На фоне 12-месячной терапии у пациентов в нашем исследовании статистически значимо снизился уровень АМГ, что свидетельствует о созревании клеток Сертоли, однако добиться значимого повышения уровня ингибина B не удалось, по-видимому, по причине недостаточной продолжительности применяемой низкодозированной терапии.

Таким образом, терапия препаратами гонадотропинов имеет такие ограничения, как отсроченный и недостаточный ответ на терапию при тяжелых формах заболевания, что приводит к невыраженной андрогенизации пациентов и ухудшению диспропорции сегментов тела. Требуется проведение дальнейших исследований для определения возраста инициации терапии, показаний к ее назначению и тактики наблюдения за такими пациентами в период лечения.

## ОГРАНИЧЕНИЯ ИССЛЕДОВАНИЯ

В ходе исследования могли возникнуть смещения результатов по причине недостаточного объема выборки в связи с низкой встречаемостью заболевания.

## ЗАКЛЮЧЕНИЕ

Прогрессия полового развития, достоверное повышение уровня тестостерона и снижение уровня АМГ на фоне лечения свидетельствуют об эффективности терапии гонадотропинами вследствие созревания клеток Сертоли.

Однако терапия гонадотропинами в течение года не позволила значимо увеличить объем яичек и уровень ингибина В, что, по-видимому, обуславливается недостаточной продолжительностью исследования, а также применением препаратов гонадотропинов в низких дозах. Необходимы дальнейшие исследования для изучения вопроса, позволит ли применение высоких доз или большей кратности инъекций добиться лучшего ответа на терапию.

## ДОПОЛНИТЕЛЬНАЯ ИНФОРМАЦИЯ

Информация о конфликте интересов. Авторы декларируют отсутствие конфликта интересов.

Информация о финансировании. Работа выполнена при содействии Фонда поддержки и развития филантропии «КАФ».

Участие авторов. Кокорева К.С., Чугунов И.С. — поисково-аналитическая работа и подготовка финальной версии статьи; Карева М.А., Безлепкина О.Б. — редактирование текста, внесение ценных замечаний. Все авторы одобрили финальную версию статьи перед публикацией, выразили согласие нести ответственность за все аспекты работы, подразумевающую надлежащее изучение и решение вопросов, связанных с точностью или добросовестностью любой части работы.
